# Repeated intravesical platelet-rich plasma injections alleviate symptoms via T-cell modulation and mitochondrial dysfunction in non-ulcer interstitial cystitis/bladder pain syndrome

**DOI:** 10.1038/s41598-026-52272-6

**Published:** 2026-05-18

**Authors:** Weilin Fang, Lin Liu, Xin Song, Jin Huang, Rong Lv, Ziwei Li, Tingting Lv, Zhikang Cai, Zhong Wang, Conghui Han, Jianwei Lv

**Affiliations:** 1 Department of Urology, Pudong Gongli Hospital, Shanghai University of Medicine & Health Sciences, No. 219 Miaopu Road, Pudong New Area, Shanghai, 200135 China; 2 Department of Urology, Xuzhou Central Hospital, No. 199 Jiefang South Road, Quanshan District, Xuzhou, 221009 Jiangsu Province China

**Keywords:** Interstitial cystitis/bladder pain syndrome, Platelet-rich plasma, T cell immune dysfunction, Diseases, Immunology, Medical research, Urology

## Abstract

Repeated intravesical injections of autologous platelet-rich plasma (PRP) have shown promise in alleviating symptoms of non-ulcer interstitial cystitis bladder pain syndrome (IC/BPS), but the underlying mechanisms remain unclear. In this single-center prospective study, 80 patients received four monthly PRP injections, with outcomes assessed by symptom scales, urodynamic parameters, and immune indices in urine and serum. PRP significantly reduced 24-h micturition frequency, numeric rating scale (NRS), O’Leary, pelvic pain and urgency/frequency patient symptom scale (PUF), and self-rating anxiety scale (SAS) scores at post-treatment follow-ups (all *p* < 0.05), while bladder capacity and voided volume remained unchanged. Serum and urinary inflammatory, iron metabolism, and oxidative stress markers were not significantly altered. PRP improved T-lymphocyte mitochondrial metabolic status, reducing CD4^+^ and CD8^+^ T-cell mitochondrial mass and decreasing CD8^+^ effector memory (Tem) T-cell counts, CD8^+^ Tem-MMP^low^, and CD8^+^ PD-1^+^ Tem counts after the fourth injection (all *p* < 0.05). These immunological parameters positively correlated with symptom severity. Baseline NRS > 4 was associated with worse baseline profiles and selective post-treatment improvements, whereas global response assessment (GRA) stratification showed no significant differences. These findings indicate that repeated intravesical PRP alleviates IC/BPS-related pain and urinary symptoms primarily by reversing T-cell exhaustion and enhancing mitochondrial metabolic status, thus highlighting T-cell immunometabolic modulation as a key therapeutic mechanism.

**Supplementary Information:**

The online version contains supplementary material available at 10.1038/s41598-026-52272-6.

## Introduction

The diagnosis of interstitial cystitis/bladder pain syndrome (IC/BPS) is established when patients present with bladder pain, pressure, tenderness, or discomfort without evidence of other underlying pathological conditions^1^. Urothelial dysfunction caused by IC/BPS may lead to reduced urothelial barrier function, injury to the mucosa, and increased permeability. As a consequence of IC/BPS, there may be prolonged inflammation, over-expression of nociceptors and a resulting onset of somatic functional syndromes which adversely impact the quality of life of IC/BPS patients^2^. Treatment with intravesical hyaluronic acid instillation and also oral pentosan polysulfate are effective for some symptoms. Nevertheless, the failure to repair mucosal barrier damage will continue to limit long-term efficacy. The restriction surrounding the treatment highlights the urgent need for new reparative strategies.

Growth factor-rich platelet-rich plasma (PRP) can help not only in wound healing of damaged epithelium but also in cell proliferation and differentiation^3^. There is considerable interest in PRP because of its special regenerative medicine ability^4–6^. Early research has demonstrated that patients with IC/BPS experience less pain and less frequent urination when PRP is injected submucosally into the bladder^7^. This implies that it is a therapeutic option that is both safe and effective^8^. PRP may function mechanistically by altering the body’s inflammatory processes. Its association with clinical outcomes, which are represented by tumor necrosis factor-α levels, is evidence of this. PRP’s capacity to regulate inflammatory pathways is further demonstrated by its ability to lessen cyclophosphamide-induced cystitis in rats. Compared to other IC/BPS treatment methods, PRP has a better safety profile^8–10^. Moreover, PRP preparation is also far less expensive than neuromodulator devices, and repeated injections can be performed in an outpatient setting, reducing hospitalization and associated patient burden. Notably, despite the evidence supporting PRP’s therapeutic potential, the current data is still limited because of the various research into small sample sizes. Furthermore, it is still necessary to clarify the basic mechanisms of action.

On mucosal and cutaneous surfaces, T lymphocytes are crucial^11,12^. Fresh insights into the function of T cells in bladder disorders are now possible because of newer technologies like single-cell RNA sequencing and spatial transcriptomics^13^. Research indicates that T lymphocytes play a critical role in mediating bladder inflammation in both human patients with interstitial cystitis and animal models that mimic this urological condition^14^. When IC/BPS patients undergo cystoscopy, the bladder submucosa often shows lymphocytic infiltration, and in some cases, Hunner’s ulcers are seen, suggesting T cell participation in local tissue damage^15^. According to recent studies, the bladder microenvironment of IC/BPS patients exhibits aberrant expressions of immune checkpoint molecules such as PD-1 and CTLA-4^16^. Inflammatory responses are reduced when this pathway is pharmacologically blocked with the CXCR5 antagonist TAK-779, suggesting a possible mechanism of immunological dysregulation caused by T cell hyperactivation in IC/BPS^16^. T cell populations that are hyperactive or metabolically dysregulated in IC/BPS may increase nociceptive signals and impair bladder function. According to recent research, T cell activation, proliferation, and differentiation are significantly influenced by mitochondrial metabolism^17,18^. A key hub for regulating T cell metabolism, mitochondria also affect the fate and function of CD8^+^ T cells. Improved ROS generation is linked to improved effector function in CD8^+^ T cells with higher mitochondrial membrane potential^19^. Notably, two crucial aspects of IC/BPS are oxidative stress and chronic inflammation, both of which are mediated by mitochondria^20,21^. It has been demonstrated that preventing mitochondrial damage can regulate the course of inflammation^22,23^. When considered collectively, T cells are essential to the pathophysiological mechanisms of bladder disorders, especially IC/BPS. Notably, there is still a dearth of studies examining whether T cell-mediated immunomodulation contributes to the reduction of pain and frequency of micturition in IC/BPS patients with intravesical submucosal injection of PRP.

In addition to examining the dynamic changes in T-cell modulation and mitochondrial dysfunction during treatment that underlie PRP therapeutic efficacy, this study concentrated on the reduction of pain and micturition frequency in non-ulcer IC/BPS patients after intravesical submucosal injection of PRP. Low mitochondrial membrane potential (MMP^low^) and mitochondrial mass (MM) are two indicators of immune cell mitochondrial function that we used in our study using a novel immunofluorescence technique. This work extends our knowledge of the immunological foundations of PRP therapy for non-ulcer IC/BPS by offering potential targets for the development of novel therapeutic approaches against T cell immune dysfunction to improve patient symptoms and quality of life.

## Methods

### Ethical statement

This study underwent ethical review and approval by the Ethics Committee of Gongli Hospital of Shanghai Pudong New Area (approval number: GLYY1s2023-025) and was conducted in strict accordance with the principles of the World Medical Association Declaration of Helsinki. This study was retrospectively registered at the Chinese Clinical Trial Registry (ChiCTR) under registration number ChiCTR2400084009 on May 9, 2024, prior to the completion of the intervention phase and before any data analysis. All participants signed a written informed consent form that elaborated on the study objectives, intravesical submucosal injection of PRP procedures, potential adverse reactions, and the right to withdraw.

### Patients recruitment

This study was a single-center, single-arm, prospective study. A total of 80 patients diagnosed with non-ulcer IC/BPS at Shanghai Pudong New Area Gongli Hospital, between September 2023 and July 2024, who had failed conventional therapies, were recruited.

Inclusion criteria were as follows^24^:

- Aged over 18 years old.
- Patients presenting with symptoms such as urinary frequency, urgency, lower abdominal pain or distension, with a duration exceeding six weeks.
- Patients previously diagnosed with non-ulcer IC/BPS by cystoscopic hydrodistension under anesthesia, or those who failed to achieve sustained or satisfactory symptom relief with empirical therapies (including oral famotidine, loratadine, intravesical hyaluronic acid instillation etc.).
- Exclusion of other diseases via urodynamic and imaging examinations.
- Patients with a history of receiving ≥ 3 types of treatments, including but not limited to lifestyle modifications, oral medications (famotidine, loratadine, deanxit, pregabalin), acupuncture-physiotherapy, intravesical hyaluronic acid instillation, botulinum toxin intradetrusor injection, cystoscopic hydrodistension, sacral neuromodulator implantation, etc., without symptomatic improvement.
- Patients were willing to participate in the study and had signed the informed consent form.

Exclusion criteria were as follows:

- Patients in the active phase of urinary tract infection.
- Patients receiving anticoagulants or antiplatelet therapy.
- T-cell lymphoma complicated with systemic immune disorders^25^.
- Platelet count < 100 × 10^9^/L and hemoglobin < 100 g/L^26^.
- Patients with severe cardiovascular and cerebrovascular diseases.
- Patients with other medical conditions precluding platelet apheresis.
- Patients were unwilling to participate in the study.

### Operation methods

For each subject, a pre-blood collection assessment was conducted before the first intravesical PRP injection, encompassing complete blood count (CBC) testing, coagulation function evaluation, and inquiry into a history of coagulation dysfunction disorders. For subsequent blood collections (2nd, 3rd, and 4th administrations), CBC testing alone was performed prior to the procedure. PRP was prepared using the Arthrex ACP™ Double Syringe System (Arthrex, USA, ABS-10014/ABS-10014-E) in combination with a Drucker HORIZON 24 FLEX-AH centrifuge (Drucker Medical, USA, 00,389-129-000 K), following the manufacturer’s standardized protocol. Briefly, approximately 15 mL of venous blood was collected via a 21G butterfly needle, with optional use of 1.5 mL anticoagulant if injection was delayed beyond 30 min. Samples were centrifuged at 1500 rpm for 5 min to separate the plasma fraction. The autologous conditioned plasma, or upper plasma layer, was moved into the inner syringe and made ready for injection. The platelet concentration ratio in relation to whole blood was computed and utilized as a quality control indication after platelet counts were obtained from a 0.5 mL sample.

Transurethral intravesical submucosal injection of PRP was performed. Intravenous anesthesia was administered, and the patient was placed in the lithotomy position. An F21 cystoscope was introduced without routine hydrodistension, and injection was initiated once a clear visual field was achieved. A bladder injection needle was inserted, and 20 injection sites were selected as uniformly as possible throughout the cystoscopic field of view. Approximately 0.5 mL of PRP was injected at each site. Following injection, normal saline was instilled into the bladder at a pressure of 80 cm water column. Infusion was halted when the fluid level in the infusion set stabilized. The saline was immediately drained, and the volume was measured, which was defined as the intraoperative bladder capacity (IBC).

After the surgery, a urinary catheter was indwelled for 1d. Antibiotics were administered via intravenous drip once to prevent urinary tract infections. Patients were discharged on the first or second postoperative day. Injections were performed once monthly for a total of four times. Follow-ups were conducted one month after each injection, with the final follow-up carried out three months after the fourth injection.

### Observation indicators

The global response assessment (GRA), which assessed the degree of symptomatic improvement following therapy, served as the study’s main outcome. The following were the scoring criteria: Marked deterioration is worth -3 points; moderate worsening is worth -2 points; mild worsening is worth -1 point; no relief is worth 0 points; mild improvement is worth + 1 point; moderate improvement is worth + 2 points; and marked improvement is worth + 3 points. For every case, GRA was evaluated one month after surgery. Bladder capacity, IBC, 24-h micturition frequency, maximum single voiding volume, O’Leary-sant score, numeric rating scale (NRS) score, pelvic pain and urgency/frequency patient symptom scale (PUF) score, self-rating anxiety scale (SAS) score, 12 serum inflammatory factors, 12 urinary inflammatory factors, and iron metabolism assessment indices were the secondary endpoints. To account for variations in urine concentration, urinary inflammatory factor levels were normalized to urinary creatinine measured in the same urine sample and expressed as creatinine-adjusted values, calculated using the following formula^27^: ${Cytokine}_{normalized}=\frac{Cytokine concentration (pg/mL)}{Urine creatinine(mg/dL)}$. Bladder capacity, and 24-h micturition frequency were obtained from patient-reported bladder diaries completed prior to each treatment session. Maximum single voiding volume was determined during hospitalization via uroflowmetry. In addition, IBC was measured under general anesthesia, with intravesical pressure dynamically maintained below 80 cmH_2_O. This measurement minimizes the influence of urine-induced sensory stimulation and pain in awake IC/BPS patients and is therefore considered to reflect the bladder’s physical maximal capacity. Peripheral blood CD4^+^ and CD8^+^ T-cell subsets were identified by flow cytometry (UBBio, DiagCyto6C2L, China) using CD45RA, CD62L, and PD-1 markers, while mitochondrial function was assessed by staining with MitoDye at 37 °C for 30 min, followed by analysis with NovoExpress software (Agilent, version 1.5.8).

### Statistical analysis

Statistical analyses were performed using GraphPad Prism software (version 10.1.2). Data were presented as mean ± standard deviation (SD) or median (interquartile range). In cases where the data were normally distributed and variances were homogeneous, ordinary one-way ANOVA was followed by Bonferroni’s post hoc test used to compare the differences between multitype groups. For non-normally distributed data or when variances were unequal, non-parametric tests, such as the Kruskal-Wallis test with Dunn’s post hoc test. Receiver operating characteristic (ROC) curve analysis with area under the ROC curve (AUC) was performed to assess the predictive ability of baseline NRS for treatment response. *P*-values < 0.05 were considered statistically significant.

## Results

A total of 80 non-ulcer IC/BPS patients were enrolled in this study, including 73 female and 7 male patients, with a mean age of 55.9454 (44, 64). All patients completed four repeated intravesical injections, and the final follow-up was performed three months after the fourth procedure. During the procedures involving 80 patients, five patients underwent bladder irrigation due to gross hematuria. No other patients experienced significant complications such as transient urinary retention, dysuria, or urinary tract infection. PRP preparation achieved a mean platelet concentration ratio of 1.841 ± 1.762 relative to peripheral blood, indicating effective enrichment of platelets. The median absolute platelet count in the injected PRP was 2.32×10^5^ cells/μL (based on baseline quality control data). All patients were discharged uneventfully after the procedures. Table 1 shows the baseline clinical parameters of non-ulcer IC/BPS patients undergoing PRP treatment.

**Table 1:** Baseline clinical parameters in the IC/BPS patients who underwent repeated intravesical PRP injection.

	IC/BPS (n = 80)
Sex (female/male)	n = 73/n = 7
Age (years old)	55.99 ± 12.84
Disease duration (< 6 months, 6 months to 2 years, > 2 years)	n = 22/n = 28/n = 30
Pharmacotherapy (yes/no)	n = 64/n = 16
Sacral neuromodulation (yes/no)	n = 5/n = 75
Platelet concentration ratio	1.841 ± 1.762

### Injection of PRP improves pain symptoms and micturition frequency in non-ulcer IC/BPS.

**Fig. 1:**
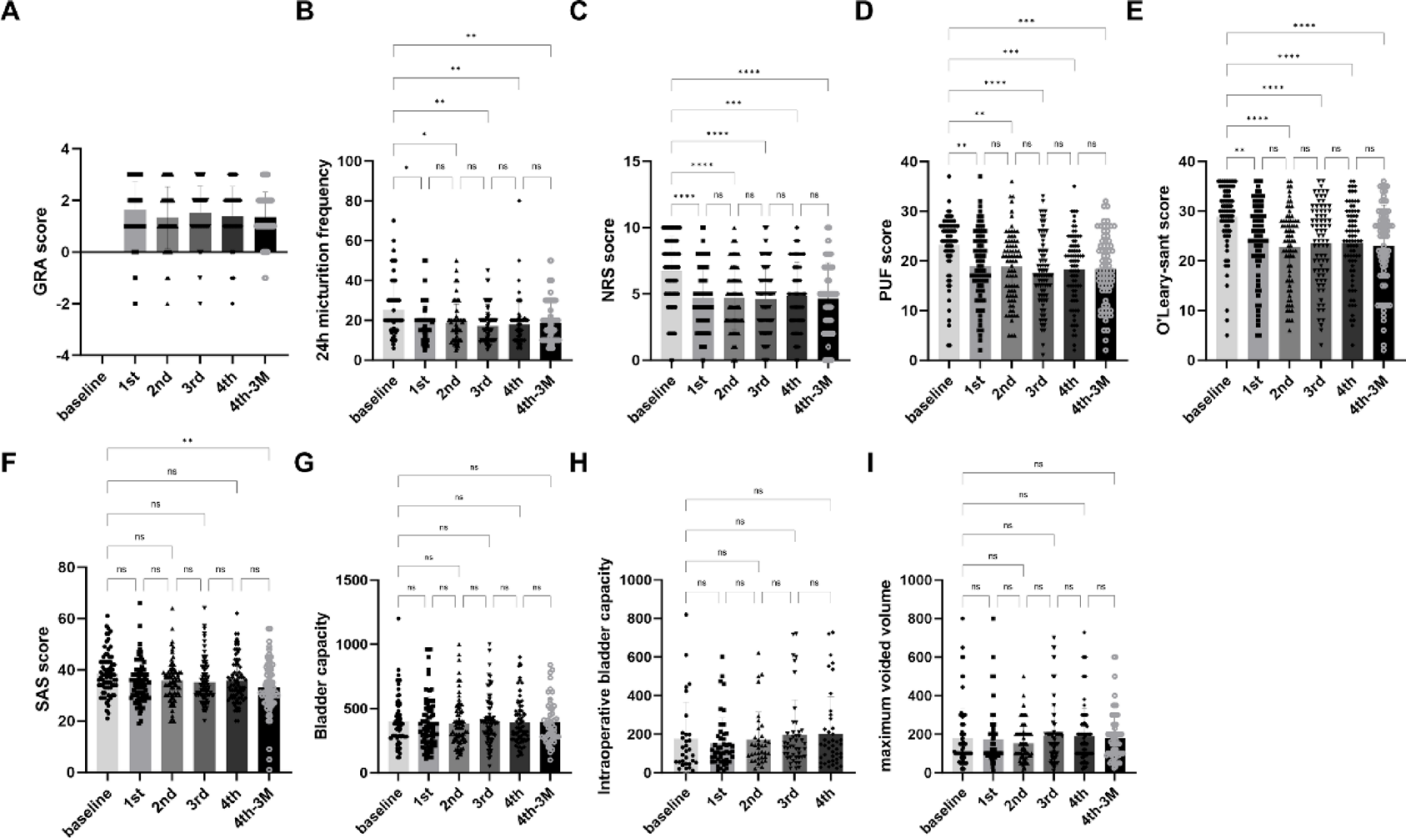
Injection of PRP improves pain symptoms and micturition frequency in non-ulcer IC/BPS. Changes in (**A**) GRA score, (**B**) 24-h micturition frequency, (**C**) NRS score, (**D**) PUF score, (**E**) O’Leary score, (**F**) SAS score, (**G**) bladder capacity, (**H**) IBC, and (**I**) maximum voided volume of IC/BPS patients after intravesical PRP injections. Statistical significance was determined by appropriate tests: *p < 0.05, **p < 0.01, ***p < 0.001, ****p < 0.0001, ns: not significant.

Figure 1 presents the comparison of observed indicators among 80 non-ulcer IC/BPS patients before treatment, after each injection, and during the follow-up three months after the fourth injection. Across the treatment course, GRA scores exhibited a progressive upward trend from the first to the fourth PRP session, followed by a slight decline in the three-month post-treatment follow-up (Fig. 1A). Trend analysis revealed a cumulative therapeutic effect of repeated PRP injections on multiple clinical outcomes. Compared with baseline, 24-h micturition frequency, NRS scores, PUF scores and O’Leary scores showed significant reduction at all post-treatment time points, including after each PRP injection and at the three-month follow-up after the fourth injection (all *p* < 0.05, Fig. 1B–E). Moreover, a significant reduction in SAS score was observed at the three-month follow-up after the fourth injection compared with baseline (*p* < 0.05, Fig. 1F). In contrast, no statistically significant changes were detected in bladder capacity, maximum voided volume, or IBC following PRP injection (*p* > 0.05, Fig. 1G-I). Furthermore, correlation analyses were performed to assess the relationships between IBC and bladder capacity. No significant correlations were found (all *p* > 0.05). These findings collectively indicate that submucosal injection of PRP was associated with significant and sustained improvements in pain intensity and urinary symptom scores.

### PRP injection does not alter inflammation cytokine and antioxidative in non-ulcer IC/BPS

Prior research has consistently revealed that IC/BPS patients exhibit a heightened level of pro-inflammatory cytokines in serum^28^. We investigated the changes in inflammatory factors in both serum and urine samples of non-ulcer IC/BPS patients following intravesical submucosal injection of PRP. Compared with baseline, no statistically significant changes were observed in any measured serum or urinary cytokines at any post-treatment time point (all *p* > 0.05). Oxidative stress has been shown to drive the pathogenesis of IC/BPS, whereas iron serves as an obligatory component in ROS production^29,30^. Thus, we investigated the changes in iron metabolism and oxidative stress-related parameters in IC/BPS patients following PRP injection. However, no statistically significant alterations were observed in these markers (all *p* > 0.05). These findings suggest that the inflammatory cytokine and antioxidative impact of PRP may be limited or delayed in non-ulcer IC/BPS.

### PRP injection modulates T cell phenotype and mitochondrial metabolic activity in non-ulcer IC/BPS

**Fig. 2:**
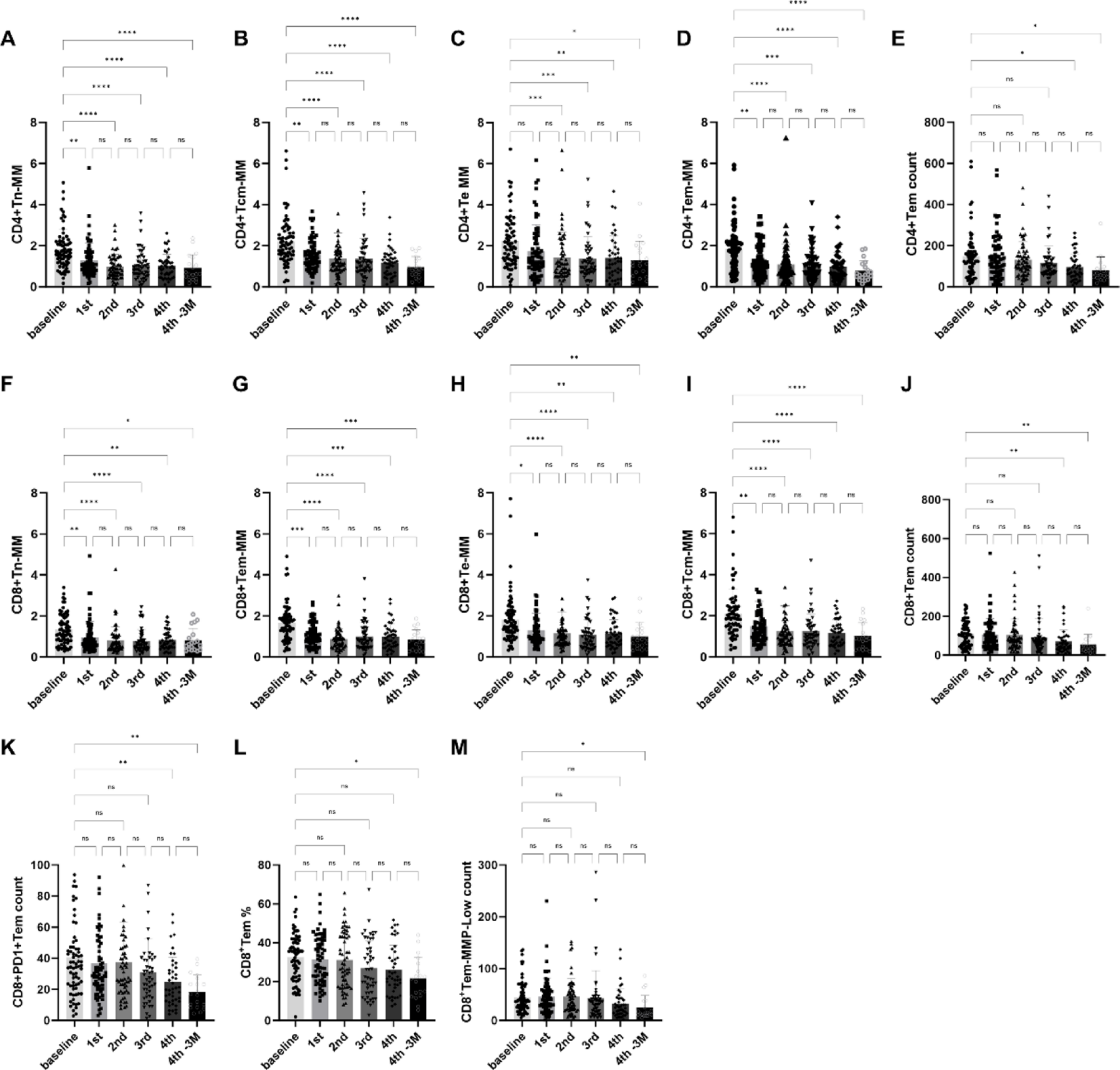
PRP injection modulates T cell phenotype and mitochondrial metabolic activity in non-ulcer IC/BPS. Changes in (**A**) CD4^+^ Tn-MM, (**B**) CD4^+^ Tcm-MM, (**C**) CD4^+^ Te-MM, (**D**) CD4^+^ Tem-MM, (**E**) CD4^+^ Tem-MM count, (**F**) CD8^+^ Tn-MM, (**G**) CD8^+^ Tcm-MM, (**H**) CD8^+^ Te-MM, (**I**) CD8^+^ Tem-MM, (**J**) CD8^+^ Tem count, (**K**) CD8^+^ PD1+ Tem count, (**L**) CD8+ Tem %, and (**M**) CD8+ Tem-MMP^low^ of IC/BPS patients after intravesical PRP injections. Statistical significance was determined by appropriate tests: *p < 0.05, **p < 0.01, ns: not significant.

Previous studies have shown that immune cell activation and mitochondrial dysfunction are closely related to IC/BPS^21,31^.To evaluate the impact of PRP submucosal bladder injection on T cell phenotype and mitochondrial function status in non-ulcer IC/BPS patients, we detected the numbers of T lymphocyte subsets and their MM and MMP^low^ before and after the first three PRP injections. Compared to pre-injection baselines, MM-associated parameters of CD4^+^ T cells, including naive CD4^+^ T cells (CD4^+^ Tn-MM), effect CD4^+^ T cells (CD4^+^ Te-MM), central memory CD4^+^ T cells (CD4^+^ Tcm-MM), and effector memory CD4^+^ T cells (CD4^+^ Tem-MM), exhibited significant decreases at all post-treatment time points (all *p* < 0.05, Fig. 2A–D). A significant reduction of CD4^+^ Tem counts were detected only after the fourth PRP injection and at the three-month follow-up following the fourth injection (both *p* < 0.05, Fig. 2E). Similarly, CD8^+^ T-cell mitochondrial mass–related parameters, including CD8^+^ Tn-MM, CD8^+^ Te-MM, CD8^+^ Tcm-MM, and CD8^+^ Tem-MM, were markedly reduced at all post-treatment time points compared with baseline (all *p* < 0.05, Fig. 2F–I). Regarding effector memory populations, CD8^+^ Tem counts and CD8^+^ PD-1^+^ Tem counts showed significant decreases only after the fourth injection and at the three-month follow-up (all *p* < 0.05, Fig. 2J–K). Additionally, CD8^+^ Tem percentage and CD8^+^ Tem-MMP^low^ counts were significantly reduced exclusively at the three-month follow-up after the fourth PRP injection (all *p* < 0.05, Fig. 2L–M). These results indicate that PRP improves the mitochondrial function status of T lymphocytes.

**Fig. 3:**
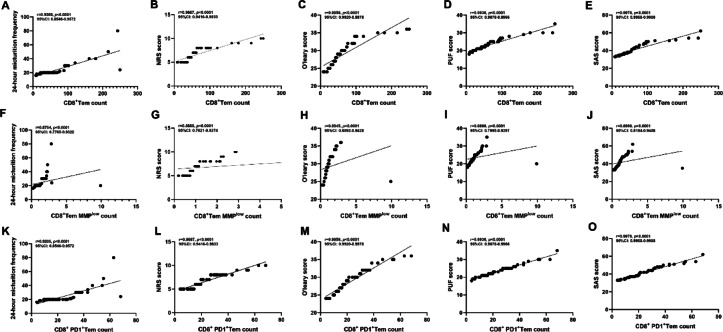
CD8^+^ Tem subsets demonstrated significant positive correlations with clinical features. Scatter plots with linear regression lines show significant positive correlations of CD8^+^ Tem counts with (**A**) 24-h micturition frequency, (**B**) NRS pain score, (**C**) O’Leary score, (**D**) PUF score, (**E**) SAS score, CD8^+^ Tem-MMP^low^ counts with (**F**) 24-h micturition frequency, (**G**) NRS pain score, (**H**) O’Leary score, (**I**) PUF score, (**J**) SAS score, and CD8^+^ PD-1^+^ Tem counts with (**K**) 24-h micturition frequency, (**L**) NRS pain score, (**M**) O’Leary score, (**N**) PUF score, and (**O**) SAS score,. Each plot includes Pearson correlation coefficient (r), *p*-value, and 95% confidence interval (95%CI).

Correlation analysis between CD8^+^ Tem count, CD8^+^ Tem-MMP^low^ counts, and CD8^+^ PD-1^+^ Tem counts and important clinical parameters that showed notable treatment-related changes were carried out in order to further investigate the relationship between PRP-induced T-cell modulation and clinical symptom changes in IC/BPS patients. The findings showed that all five assessed clinical features—24-h micturition frequency, NRS pain score, O’Leary score, PUF score, and SAS score—showed significant positive correlations with CD8^+^ Tem count, CD8^+^ Tem-MMP^low^ counts, and CD8^+^ PD-1^+^ Tem counts (all *p* < 0.05, Fig. 3A–O). According to these findings, PRP therapy may reduce inflammation in non-ulcer IC/BPS by reestablishing T-cell function and lowering excessive activation.

### NRS stratification influences therapeutic outcomes of PRP in non-ulcer IC/BPS

**Fig. 4:**
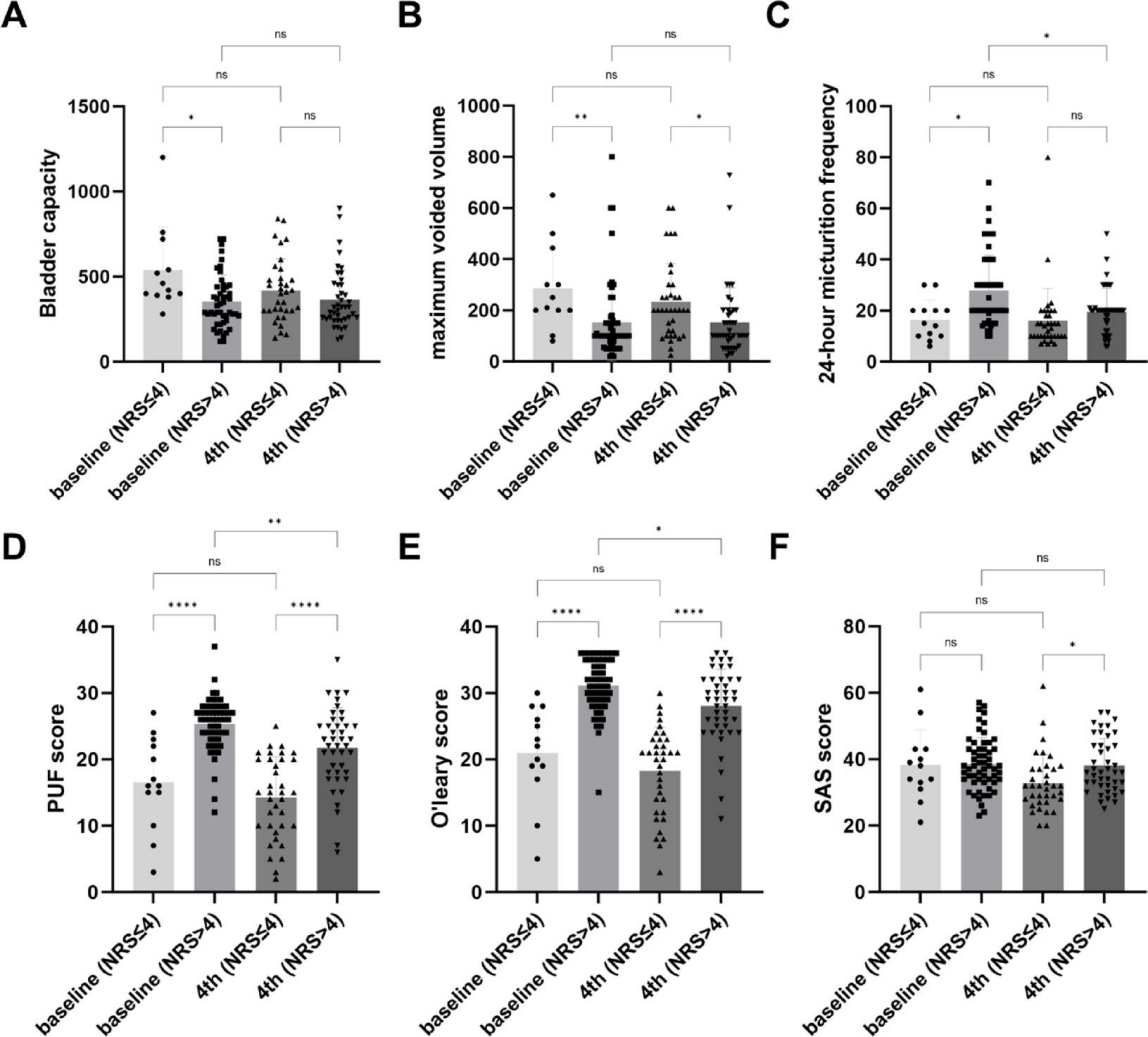
NRS stratification influences therapeutic outcomes of PRP in non-ulcer IC/BPS. Stratified analysis of (**A**) bladder capacity, (**B**) maximum voided volume, (**C**) 24-h micturition frequency, (**D**) PUF score, (**E**) O’Leary score, and (**F**) SAS score in IC/BPS patients based on NRS score. Groups are defined as: baseline (NRS ≤ 4), baseline (NRS > 4), 4th (NRS ≤ 4), 4th (NRS > 4) representing different pain intensity stratifications before and after fourth PRP treatment. Statistical significance was determined by appropriate tests: *p < 0.05, **p < 0.01, ****p < 0.0001, ns: not significant.

It is generally accepted that moderate to severe pain is indicated by an NRS score greater than 4^32^. In order to determine the impact of PRP therapy on pain relief and symptom improvement in patients with non-ulcer IC/BPS, patients were stratified based on their baseline NRS scores into two groups: those with an NRS score > 4 and those with an NRS score ≤ 4. Different patterns were found when the analysis was stratified by NRS score. Patients with an NRS score greater than 4 had considerably worse clinical profiles at baseline than patients with an NRS score less than 4. In particular, bladder capacity, maximum voided volume, 24-h micturition frequency, PUF score, and O’Leary score all showed significant intergroup differences (all *p* < 0.05, Fig. 4A–E). Maximum voided volume, PUF score, O’Leary score, and SAS score were among the parameters that showed significant differences between the two NRS subgroups after the fourth PRP injection (all *p* < 0.05, Fig. 4B, D–F). PRP treatment was linked to significant post-treatment improvements in 24-h micturition frequency, PUF score, O’Leary score, and SAS score among patients with baseline NRS > 4, according to within-group longitudinal analyses (all *p* < 0.05, Fig. 4C–F). Furthermore, following the fourth PRP injection, patients were divided into two groups based on their GRA scores: GRA < 2 and GRA ≥ 2. However, after PRP treatment, no statistically significant differences in clinical parameters were found between the two GRA subgroups or within each subgroup (all *p* > 0.05). To further evaluate whether baseline NRS could serve as a reliable predictor of marked clinical improvement (defined as GRA ≥ 2), a ROC curve analysis was performed. The AUC was 0.536, indicating poor discriminative ability (Figure S1). These results suggest that baseline NRS alone is insufficient to reliably predict treatment response. Collectively, these findings suggest that baseline NRS score exerts a stratifying modulatory effect on symptom burden and selective treatment-associated improvements following PRP therapy in non-ulcer IC/BPS patients. In contrast, GRA-based stratification did not reveal significant differences in baseline demographic characteristics.

## Discussion

Intravesical submucosal injection of PRP decreased pain scores and micturition frequency in IC/BPS patients, albeit the precise mechanism of action is yet uncertain. Through submucosal injection of autologous PRP for refractory non-ulcer IC/BPS, this study elucidated the possible role of PRP in improving pain, micturition frequency, and modifying T cell immunological homeostasis. This offered fresh perspectives for improving treatment plans or guiding immunotherapies in non-ulcer IC/BPS.

Our findings demonstrated that intravesical PRP injections are associated with significant reductions in pain and particular urine symptoms in non-ulcer IC/BPS patients because of their remarkable capacity to alleviate discomfort. This aligns with previous relevant reports^33^. The lack of significant improvement in intraoperative and daily-life bladder capacity following PRP treatment may be explained by the underlying anatomical and neuroplastic changes commonly seen in IC/BPS^34^. In chronic phases of the disease, persistent urothelial damage, fibrosis, and detrusor overactivity may impair the bladder’s morphological or functional capacity even in the presence of symptomatic alleviation^35^. Notably, urinary frequency and PUF score significantly decreased following PRP treatment, despite the maximum bladder capacity remaining unchanged. Given that voiding frequency is typically thought to be closely related to bladder capacity, this finding seems counterintuitive. PRP treatment may have extended the storage phase and decreased the frequency of voiding by raising the threshold volume that causes urgency sensations. The primary pathophysiological feature of IC/BPS is the disruption of the urothelial barrier, which permits abnormal stimulation of submucosal afferent nerves by urinary solutes, resulting in increased bladder sensation and early urgency^36^. In this case, the results of PRP therapy probably show that the damaged urothelial mucosa has been structurally and functionally repaired, reducing the abnormal stimulation of the bladder wall caused by urine. According to recent spatial transcriptome research, immune cells are enriched close to the urothelium and interact with sensory networks^13^. These results show that rather than depending on anatomical expansion of bladder capacity, PRP may mainly reduce symptoms by modifying local sensory-neural pathways. Although intravesical PRP injections did not result in immediate changes in anxiety levels, a significant reduction in SAS scores was observed at the three-month follow-up after completion of the PRP treatment course. This delayed improvement suggests that psychological benefits may occur secondary to sustained symptom relief, rather than as a direct effect of PRP therapy. Given that anxiety is known to modulate pain perception and symptom severity in IC/BPS^37^, These findings indicate that PRP may primarily target somatic and sensory mechanisms, while adjunctive psychological or behavioral interventions may further enhance long-term outcomes, particularly in patients with comorbid anxiety.

Since inflammation has been connected to the pathogenesis and evolution of IC/BPS, targeting inflammatory cytokines in IC/BPS patients has become a promising therapeutic strategy^38^. In contrast to previous reports, no significant baseline-adjusted changes in blood or urine cytokines were observed in this study^39,40^. Variations in test sensitivity, patient characteristics, PRP production methods, and sample periods between studies may account for this discrepancy^7^. Furthermore, it is believed that PRP primarily affects the bladder milieu, and systemic or urine cytokine measures may not accurately reflect such localized immune regulation. Therefore, the lack of discernible cytokine changes in the current cohort emphasizes the temporal and geographical heterogeneity of PRP-induced inflammatory responses in IC/BPS rather than necessarily contradicting previous observations.

Because ROS affect bladder function through a variety of molecular processes, oxidative stress has been linked to the pathophysiology of IC/BPS^29^. Loss of Wnt/β-catenin signaling in IC/BPS promotes oxidative stress-induced ferroptosis and exacerbates bladder injury by enabling NF-κB-driven inflammatory cytokines to activate the JAK-STAT pathway^41^. Effective host iron sequestration can dramatically lower ROS synthesis, hence reducing inflammation, as iron is a necessary component for ROS production^30^. This approach may be a useful therapeutic strategy to improve the prognosis and quality of life for people with IC/BPS. Although it has been proposed that PRP indirectly modifies redox balance through immunoregulatory or tissue-repair-related pathways^42^, circulating or urine iron-related indicators may not adequately capture these effects since they may be highly localized inside the bladder milieu. Therefore, the role for oxidative stress regulation at the tissue level is not ruled out by the absence of appreciable changes in iron metabolism parameters. Instead, it implies that PRP-associated clinical benefits are unlikely to be mediated by overt modulation of systemic iron availability or global oxidative stress responses.

Recent data further supports PRP’s efficacy in treating IC/BPS^8^. Variations in PRP preparation methods, platelet concentrations, and injection schedules may account for some of the variation in results across studies^37^. Notably, our study expands on earlier clinical research by incorporating comprehensive immunophenotypic and mitochondrial function analyses of T cell subsets. Our findings suggest that PRP injection may exert an immunological remodeling effect on T cells in non-ulcer IC/BPS, characterized by coordinated alterations in T-cell subset composition and mitochondrial state, rather than inducing generic immune suppression. One plausible explanation is that intravesical PRP alleviates bladder mucosal inflammation and epithelial barrier dysfunction, thereby reducing persistent antigenic stimulation originating from the bladder microenvironment, which in turn reshapes systemic T-cell activation states. In addition, PRP-derived bioactive factors may exert indirect systemic effects through circulation or neuroimmune signaling pathways. However, no significant changes were observed in circulating or urinary inflammatory cytokines in our cohort, suggesting that PRP may primarily exert immunomodulatory effects through cellular reprogramming rather than broad suppression of soluble inflammatory mediators. Prior single-cell and tissue-level analyses have revealed reduced CD4^+^ Te and CD8^+^ Tem cells together with improved mitochondrial activity, suggesting that PRP alleviates symptoms via gradual immunometabolic reprogramming of T cells^13^. A decrease in mitochondrial autophagy in peripheral blood lymphocytes can lead to an abnormal increase in MM, which accumulates ROS and results in mitochondrial dysfunction^43,44^. An increase in MMP^low^ is indicative of depolarization accumulation, which also leads to mitochondrial dysfunction. Mitochondrial dysfunction is known to be a characteristic of chronic inflammation-induced T cell exhaustion, where continuous antigenic stimulation leads to metabolic insufficiency and decreased immune surveillance^45^. Notably, fatigued T cells have larger, frequently depolarized mitochondria. In this context, the observed improvement in mitochondrial parameters following PRP treatment may reflect a reduction in chronic inflammatory stimulation and restoration of mitochondrial quality control in T cells. These findings raise the possibility that PRP may restore T cell bioenergetics and reduce metabolic stress related to chronic immune activation by enhancing mitochondrial function status, as demonstrated by consistent reductions in MM-related parameters across multiple CD4^+^ and CD8^+^ T-cell subsets at all post-treatment time points. Crucially, rather than an instantaneous immune reaction after a single treatment, the widespread and time-dependent decrease in these mitochondrial parameters points to a cumulative regulatory effect of repeated PRP injections. This temporal trend is consistent with the clinical observation that symptom alleviation varies during subsequent PRP sessions. Moreover, enhanced mitochondrial activity after PRP was strongly associated with reduced T-cell exhaustion and concurrent improvements in pain, urinary symptoms, and anxiety. This points to an immunoregulatory route that links clinical benefit in non-ulcer IC/BPS to immunological remodeling.

NRS stratification is widely used to predict therapeutic outcomes and guide personalized treatment^32^. PRP has shown promise in lowering IC/BPS symptoms, but each patient responds differently to it^46^. Patients were classified using an NRS threshold of 4 to examine whether baseline pain intensity affects the clinical response to PRP therapy. Our study demonstrated that baseline pain severity exerted a stratifying and moderating effect on PRP outcomes. Higher baseline pain was associated with greater disease burden and a persistently less favorable symptom profile, yet these patients experienced larger absolute symptom improvements after treatment, suggesting that PRP may confer more perceptible benefits in individuals with more severe pain. This idea aligns with the clinical finding that patients with more severe symptoms might benefit more quantifiably from focused interventions^47^. All these findings point to baseline NRS as a stratification marker that moderates the effectiveness of PRP. The necessity of pain-stricken, multimodal therapeutic approaches in the treatment of IC/BPS is supported by these findings^48^. However, subsequent ROC analysis yielded a low AUC of 0.536, indicating that baseline NRS by itself cannot reliably predict which patients will achieve marked improvement (GRA ≥ 2). This suggests that a multimodal prediction model—incorporating clinical, immunological, and pain-related variables—may be necessary for individualized treatment stratification.

The present study contains several limitations that should be considered in addition to the positive outcomes. First off, the single-center, non-randomized design may limit generalizability and prevent definitive conclusions on the efficacy of PRP injections in non-ulcer IC/BPS. Second, the absence of placebo control limits the ability to distinguish PRP effects from alternative treatments or the natural course of the disease. Future research should use a multicenter, randomized controlled trial design to overcome these limitations and validate the results in various situations and demographics. Additionally, both T-cell phenotyping and mitochondrial function assessments were performed exclusively on peripheral blood. While the proposed model of bladder-periphery immunometabolic crosstalk provides a plausible framework for our findings, direct evidence of local T-cell modulation within the bladder submucosa remains lacking. Future studies incorporating bladder biopsies with tissue-resident T-cell profiling and cellular trafficking analyses are required to validate this mechanistic link. Moreover, mitochondrial function was assessed solely using surrogate markers such as mitochondrial mass and membrane potential. While these parameters are widely used to infer metabolic status in T cells, they do not constitute direct functional proof. Future studies employing dynamic metabolic assays, such as oxygen consumption rate and ATP production, are required to conclusively demonstrate improved mitochondrial function. Finally, no formal a priori sample size calculation was performed. Because this was a prospective exploratory study and reliable preliminary effect sizes for PRP-related T-cell mitochondrial changes in IC/BPS were unavailable, the sample size was determined by feasibility. Therefore, the statistical power of the study remains uncertain, and the findings should be interpreted as hypothesis-generating rather than confirmatory.

## Conclusion

This study has demonstrated that repeated intravesical PRP injections significantly improved pain and urinary symptoms in non-ulcer IC/BPS, primarily via modulation of effector/effector memory T cells, and mitochondrial function status restoration, rather than direct oxidative stress alteration. Despite its limitations, these findings provide critical evidence for developing immunoregulation-based individualized therapeutic strategies. Future research integrating multi-omics technologies and clinical translational studies is needed to further identify the precise action targets of PRP in non-ulcer IC/BPS.

## Supplementary Information

Below is the link to the electronic supplementary material.

Supplementary Material 1

## Data Availability

The datasets used and/or analyzed during the current study are available from the corresponding author.

## References

[1] Hanno, P. M., Erickson, D., Moldwin, R. & Faraday, M. M. Diagnosis and treatment of interstitial cystitis/bladder pain syndrome: AUA guideline amendment. *J. Urol.* **193**, 1545–1553 (2015). 25623737 10.1016/j.juro.2015.01.086

[2] Tornic, J. & Engeler, D. Latest insights into the pathophysiology of bladder pain syndrome/interstitial cystitis. *Curr. Opin. Urol.* **34**, 84–88 (2024). 38117118 10.1097/MOU.0000000000001158

[3] Everts, P., Onishi, K., Jayaram, P., Lana, J. F. & Mautner, K. Platelet-rich plasma: New performance understandings and therapeutic considerations in 2020. *Int J Mol Sci.* **21** (2020). 10.3390/ijms21207794 PMC7589810 33096812

[4] Carr, B. J. Platelet-rich plasma as an orthobiologic: Clinically relevant considerations. *Vet. Clin. North Am. Small Anim. Pract.* **52**, 977–995 (2022). 35562219 10.1016/j.cvsm.2022.02.005

[5] Barrenetxea, G. et al. Intraovarian platelet-rich plasma injection and IVF outcomes in patients with poor ovarian response: A double-blind randomized controlled trial. *Hum. Reprod.* **39**, 760–769 (2024). 38423539 10.1093/humrep/deae038

[6] Gupta, S., Paliczak, A. & Delgado, D. Evidence-based indications of platelet-rich plasma therapy. *Expert Rev. Hematol.* **14**, 97–108 (2021). 33275468 10.1080/17474086.2021.1860002

[7] Jiang, Y. H. et al. Therapeutic efficacy of intravesical platelet-rich plasma injections for interstitial cystitis/bladder pain syndrome-a comparative study of different injection number additives and concentrations. *Front. Pharmacol.* **13**, 853776 (2022). 35392571 10.3389/fphar.2022.853776 PMC8980355

[8] El Hefnawy, A. S., Hasan, M. A. A., El Sawy, E., Abdel-Razik, M. & El-Tabey, N. Intravesical instillation of platelet-rich plasma for treatment of interstitial cystitis/bladder pain syndrome: A pilot study. *Curr. Urol.* **18**, 49–54 (2024). 38505153 10.1097/CU9.0000000000000156 PMC10946635

[9] Jhang, J. F., Yu, W. R. & Kuo, H. C. Comparison of the clinical efficacy and adverse events between intravesical injections of platelet-rich plasma and botulinum toxin A for the treatment of interstitial cystitis refractory to conventional treatment. *Toxins*10.3390/toxins15020121 (2023). 36828435 10.3390/toxins15020121 PMC9961286

[10] Raisin, G. et al. Open label, pilot evaluation of the safety and efficacy of intravesical sustained release system of lidocaine and oxybutynin (TRG-100) for patients with interstitial cystitis/bladder pain syndrome, overactive bladder and patients with retained ureteral stents following endourological interventions. *Urology* **178**, 42–47 (2023). 37268171 10.1016/j.urology.2023.05.016

[11] Bousbaine, D. et al. A conserved bacteroidetes antigen induces anti-inflammatory intestinal T lymphocytes. *Science* **377**, 660–666 (2022). 35926021 10.1126/science.abg5645 PMC9766740

[12] Stolley, J. M. et al. Depleting CD103+ resident memory T cells in vivo reveals immunostimulatory functions in oral mucosa. *J. Exp. Med.*10.1084/jem.20221853 (2023). 37097449 10.1084/jem.20221853 PMC10130744

[13] Peng, L. et al. Integrating single-cell RNA sequencing with spatial transcriptomics reveals immune landscape for interstitial cystitis. *Signal Transduct. Target Ther.* **7**, 161 (2022). 35589692 10.1038/s41392-022-00962-8 PMC9120182

[14] Zhao, J. et al. Chemokine receptor 7 contributes to T- and B-cell filtering in ageing bladder, cystitis and bladder cancer. *Immun. Ageing* **21**, 33 (2024). 38762550 10.1186/s12979-024-00432-5 PMC11102276

[15] Cheng, X. F. et al. Integrated analysis of microarray studies to identify novel diagnostic markers in bladder pain syndrome/interstitial cystitis with hunner lesion. *Int. J. Gen. Med.* **15**, 3143–3154 (2022). 35342305 10.2147/IJGM.S351287 PMC8943715

[16] Zhao, J. et al. Activation of CXCL13/CXCR5 axis aggravates experimental autoimmune cystitis and interstitial cystitis/bladder pain syndrome. *Biochem. Pharmacol.* **200**, 115047 (2022). 35452631 10.1016/j.bcp.2022.115047

[17] Elhage, R. et al. Mitochondrial dynamics and metabolic regulation control T cell fate in the thymus. *Front. Immunol.* **14**, 1270268 (2023). 38288115 10.3389/fimmu.2023.1270268 PMC10822881

[18] Norton, E. G., Chapman, N. M. & Chi, H. Mitochondria and lysosomes in T cell immunometabolism. *Trends Immunol.* **46**, 635–651 (2025). 40849263 10.1016/j.it.2025.07.014 PMC12476025

[19] Sukumar, M. et al. Mitochondrial membrane potential identifies cells with enhanced stemness for cellular therapy. *Cell Metab.* **23**, 63–76 (2016). 26674251 10.1016/j.cmet.2015.11.002 PMC4747432

[20] Xu, X., Pang, Y. & Fan, X. Mitochondria in oxidative stress, inflammation and aging: From mechanisms to therapeutic advances. *Signal Transduct. Target. Ther.* **10**, 190 (2025). 40500258 10.1038/s41392-025-02253-4 PMC12159213

[21] Janev, A., Zupančič, D., Veranič, P. & Kuret, T. Oxidative stress and chronic inflammation as partners in crime in interstitial cystitis/bladder pain syndrome. *J. Innate Immun.*10.1159/000546901 (2025). 40570823 10.1159/000546901 PMC12334152

[22] Luo, F., Herrup, K., Qi, X. & Yang, Y. Inhibition of Drp1 hyper-activation is protective in animal models of experimental multiple sclerosis. *Exp. Neurol.* **292**, 21–34 (2017). 28238799 10.1016/j.expneurol.2017.02.015 PMC5484055

[23] Chen, W. et al. MitoQ attenuates brain damage by polarizing microglia towards the M2 phenotype through inhibition of the NLRP3 inflammasome after ICH. *Pharmacol. Res.* **161**, 105122 (2020). 32791262 10.1016/j.phrs.2020.105122

[24] Clemens, J. Q., Erickson, D. R., Varela, N. P. & Lai, H. H. Diagnosis and treatment of Interstitial Cystitis/Bladder Pain Syndrome. *J. Urol.* **208**, 34–42 (2022). 35536143 10.1097/JU.0000000000002756

[25] Martínez-Martínez, A., Ruiz-Santiago, F. & García-Espinosa, J. Platelet-rich plasma: Myth or reality?. *Radiologia (Engl Ed)* **60**, 465–475 (2018). 30274850 10.1016/j.rx.2018.08.006

[26] Eliasberg, C. D. et al. Complications following biologic therapeutic injections: A multicenter case series. *Arthroscopy* **37**, 2600–2605 (2021). 33872744 10.1016/j.arthro.2021.03.065

[27] Nobles, C. et al. Correlation of urine and plasma cytokine levels among reproductive-aged women. *Eur. J. Clin. Invest.* **45**, 460–465 (2015). 25721914 10.1111/eci.12428

[28] Jiang, Y. H., Peng, C. H., Liu, H. T. & Kuo, H. C. Increased pro-inflammatory cytokines, C-reactive protein and nerve growth factor expressions in serum of patients with interstitial cystitis/bladder pain syndrome. *PLoS ONE* **8**, e76779 (2013). 24146927 10.1371/journal.pone.0076779 PMC3798602

[29] Mohammad, A., Laboulaye, M. A., Shenhar, C. & Dobberfuhl, A. D. Mechanisms of oxidative stress in interstitial cystitis/bladder pain syndrome. *Nat. Rev. Urol.* **21**, 433–449 (2024). 38326514 10.1038/s41585-023-00850-y

[30] Hagn, G. et al. Iron chelation as novel treatment for interstitial cystitis. *Pharmacology* **103**, 159–162 (2019). 30695781 10.1159/000496089

[31] Wang, J. et al. Characteristic genes and immune landscape of interstitial cystitis. *PLoS ONE* **20**, e0320249 (2025). 40435311 10.1371/journal.pone.0320249 PMC12119006

[32] Snijders, G. F. et al. Treatment outcomes of a numeric rating scale (NRS)-guided pharmacological pain management strategy in symptomatic knee and hip osteoarthritis in daily clinical practice. *Clin Exp Rheumatol.* **30**, 164–170 (2012). 22325619

[33] Soliman, A., Adel, M., Elnagar, M. A., Elsonbaty, S. & Hefnawy, A. E. How intravesical platelet-rich plasma can help patients with interstitial cystitis/bladder pain syndrome: A comprehensive scoping review. *Int. Urogynecol. J.* **35**, 1735–1743 (2024). 38958727 10.1007/s00192-024-05844-x

[34] Grundy, L., Caldwell, A. & Brierley, S. M. Mechanisms underlying overactive bladder and interstitial cystitis/painful bladder syndrome. *Front. Neurosci.* **12**, 931 (2018). 30618560 10.3389/fnins.2018.00931 PMC6299241

[35] Wyndaele, M. et al. Beyond the urothelium: Interplay between autonomic nervous system and bladder inflammation in urinary tract infection, bladder pain syndrome with interstitial cystitis and neurogenic lower urinary tract dysfunction in spinal cord injury-ICI-RS 2023. *Neurourol. Urodyn.* **43**, 1283–1292 (2024). 37876314 10.1002/nau.25310

[36] Madan, R. et al. Early three-month report of amniotic bladder therapy in patients with interstitial cystitis/bladder pain syndrome. *Int. Urol. Nephrol.* **55**, 1937–1942 (2023). 37273012 10.1007/s11255-023-03652-8

[37] Kuo, H. C. Intravesical injections of autologous platelet-rich plasma for the treatment of refractory interstitial cystitis. *Low Urin. Tract Symptoms* **15**, 210–215 (2023). 37702275 10.1111/luts.12504

[38] Shah, A. M. et al. Temporally complex inflammatory networks in an animal model reveal signatures for interstitial cystitis and bladder pain syndrome phenotype. *Neurourol. Urodyn.* **42**, 1839–1848 (2023). 37587846 10.1002/nau.25267 PMC10615708

[39] Chiu, Y. C., Tsai, P. C., Jhang, J. F. & Kuo, H. C. Revealing distinct treatment mechanisms and outcome correlations in patients with interstitial cystitis/bladder pain syndrome after different bladder therapies through urinary biomarker analysis. *Int. Urol. Nephrol.* **57**, 2073–2080 (2025). 39878889 10.1007/s11255-025-04388-3

[40] Trama, F. et al. Use of intravesical injections of platelet-rich plasma for the treatment of bladder pain syndrome: A comprehensive literature review. *Antibiotics*10.3390/antibiotics10101194 (2021). 34680774 10.3390/antibiotics10101194 PMC8532598

[41] Fang, W. et al. Wnt/β-catenin signaling inhibits oxidative stress-induced ferroptosis to improve interstitial cystitis/bladder pain syndrome by reducing NF-κB. *Biochim Biophys Acta Mol Cell Res.* **1871**, 119766 (2024). 38823528 10.1016/j.bbamcr.2024.119766

[42] Peters, K. M., Killinger, K. A., Mounayer, M. H. & Boura, J. A. Are ulcerative and nonulcerative interstitial cystitis/painful bladder syndrome 2 distinct diseases? A study of coexisting conditions. *Urology* **78**, 301–308 (2011). 21703668 10.1016/j.urology.2011.04.030

[43] Yu, F. et al. Distinct mitochondrial disturbance in CD4+T and CD8+T cells from HIV-infected patients. *J Acquir Immune Defic Syndr.* **74**, 206–212 (2017). 27608061 10.1097/QAI.0000000000001175

[44] Pang, L. X. et al. The diagnostic value of mitochondrial mass of peripheral T lymphocytes in early sepsis. *Front Public Health.* **10**, 928306 (2022). 35910903 10.3389/fpubh.2022.928306 PMC9330378

[45] Yu, Y. R. et al. Disturbed mitochondrial dynamics in CD8(+) TILs reinforce T cell exhaustion. *Nat Immunol.* **21**, 1540–1551 (2020). 33020660 10.1038/s41590-020-0793-3

[46] Watanabe, T. et al. Intravesical indwelling lidocaine-releasing devices for IC/BPS. *World Acad. Sci. J.* **4**, 28 (2022).

[47] Yu, W. R., Jiang, Y. H., Jhang, J. F. & Kuo, H. C. Repeated intravesical injections of platelet-rich plasma are safe and effective in the treatment of interstitial cystitis/bladder pain syndrome. *Tzu Chi Med J.* **37**, 72–79 (2025). 39850397 10.4103/tcmj.tcmj_166_24 PMC11753520

[48] Mishra, N. N. PD05-12 why augmentation cystoplasty alone (ACA) works in intractable interstitial cystitis/bladder pain syndrome (IC/BPS). *J. Urol.* **209**, e154 (2023).

