# Supplementary figures and images for: Repeated intravesical platelet-rich plasma injections alleviate symptoms via T-cell modulation and mitochondrial dysfunction in non-ulcer interstitial cystitis/bladder pain syndrome
Source: Sci Rep. 2026 May 18;16:22462. doi: 10.1038/s41598-026-52272-6 (PMC13377193; doi:10.1038/s41598-026-52272-6)

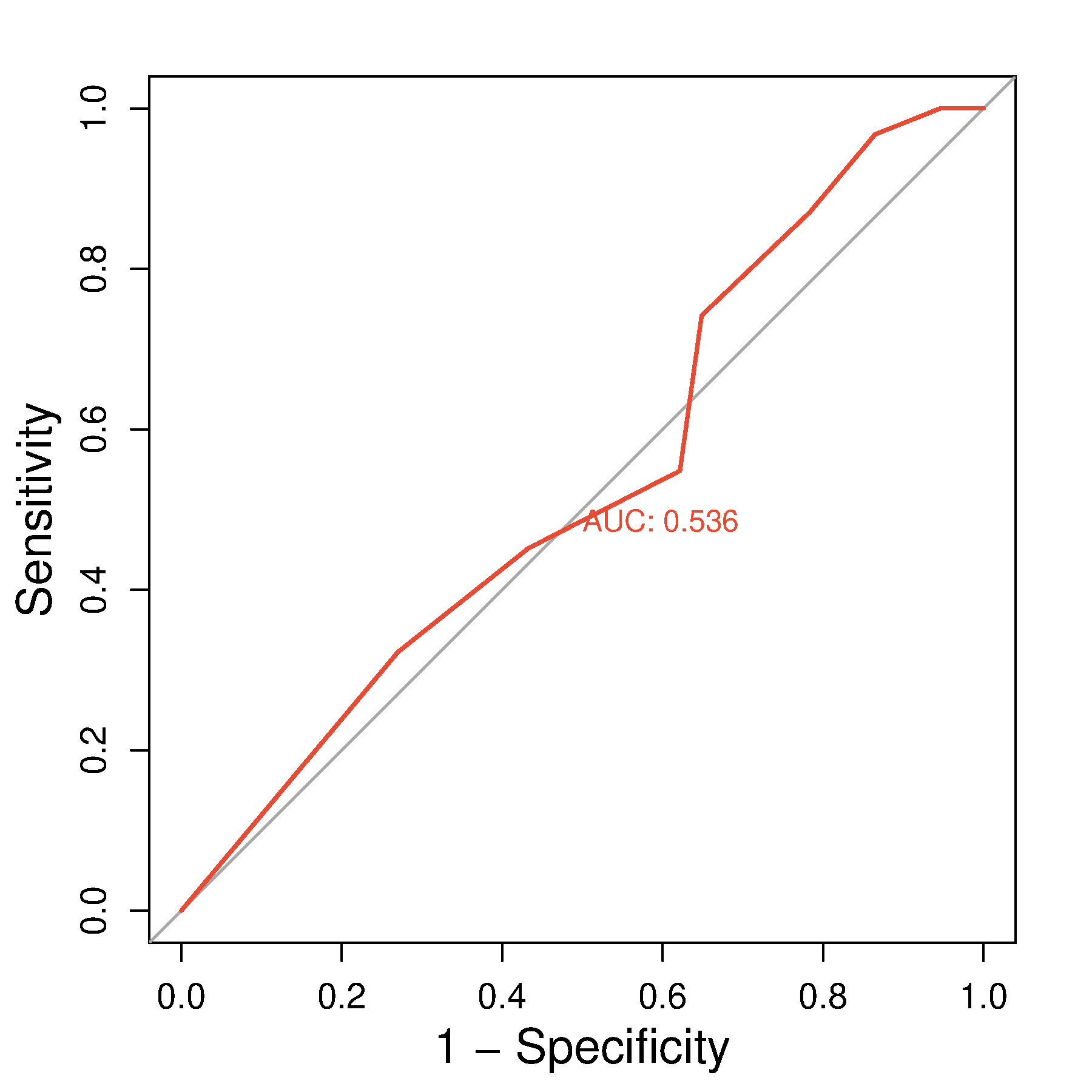

Supplement: Supplementary file 1 — Supplementary Material 1 [file 41598_2026_52272_MOESM1_ESM.tiff]
